# Efficacy of combined metformin–clomiphene citrate in comparison with clomiphene citrate alone in infertile women with polycystic ovarian syndrome (PCOS)

**Published:** 2013-06-25

**Authors:** A Ayaz, Y Alwan, MU Farooq

**Affiliations:** * Hera General Hospital, Makkah, Saudi Arabia; ** King Abdullah Medical City, Makkah, Saudi Arabia

**Keywords:** Polycystic ovaries, Metformin, Clomiphene citrate, Anovulation

## Abstract

Background. Polycystic ovary syndrome (PCOS) is the most common endocrinopathy in women and is associated with the reproductive and metabolic disorders.

Objectives. To compare the ovulation and conception rates after the treatment with Clomiphene Citrate (CC) alone and in combination with metformin in infertile patients presented with polycystic ovarian syndrome (PCOS).

Material & Methods. This randomized controlled trial of independent cases and controls was conducted in the Department of Obstetrics and Gynecology, Hera General Hospital, Makkah, Saudi Arabia, during 2008. The 42 subjects diagnosed as PCOS were divided into group A and B (21 subjects in each) for the management with CC + metformin and CC alone, respectively. Group A received 500mg of metformin continuously, three times a day from the first cycle, for 6 months or until the pregnancy was confirmed. In both groups, CC was started with a dose of 50 mg from day-2 until day-6 of the menstrual cycle. The dose of CC was increased to 100 mg in the second and 150 mg in the third cycle, and then 150 mg remained for the rest of three cycles. With ovulation, the dose of CC was unaltered in both groups. Data were analyzed by using SPSS version 16.

Results. More than 50% of the females in both groups had a body mass index of >25. Group A achieved a higher rate of regular cycles, ovulation success, and conception than group B (71.4% vs. 38.1%; p=0.03), (76.2% vs. 38.1%; p=0.021) and (66.6% vs. 28.6%, p=0.01), respectively.

Conclusion. Management with metformin + CC increased the ovulation and conception rates.

## Introduction

Polycystic ovary syndrome (PCOS) is a heterogeneous disorder with an incidence of 4–7% among reproductive aged women. It is characterized by chronic anovulation, hyperandrogenism, and it is the most frequent endocrinopathy in women [**1**]. It is commonly linked with insulin resistance and compensatory hyperinsulinemia [2,3]. Ovarian hyperandrogenism and chronic anovulation are highly linked with hyperinsulinemia but usually weight loss in obese women with PCOS leads to a decrease in insulin and androgen levels with an obvious improved fertility outcome [**4,5**]. CC is the first-line therapy for ovulation induction in these patients but it still has a high failure rate. However, patients have an increased risk of ovarian cancer in case its usage is prolonged for more than six cycles [**6,7**]. 

 A number of pharmacological agents especially metformin has been used to amplify the physiological effect of weight loss that inhibits the production of glucose in liver and augments the sensitivity of peripheral tissue to insulin, and decrease insulin secretion [**8**]. It has been shown that metformin improved hyperandrogenism and gonadotropin secretary abnormalities in women with PCOS and restored menstrual cycle and fertility [**9**].

 To the best of our knowledge and understanding, studies done on this topic are scarce in Saudi Arabia so, we set out the objectives of this study to compare the effects of CC alone and in combination with metformin in females presenting with PCOS.

## Material and Methods 

This randomized controlled trial of independent cases and controls with one control per case was conducted in the Department of Obstetrics and Gynecology, Hera General Hospital, Makkah, Saudi Arabia, during 2008. 

 The sample size was determined according to Jones et al. criteria [**10**], because, according to Vandermolen et al., in 42 subjects the ovulation failure rate of (P1=73%) was indicated in the group treated with CC alone and (P2=25%) for the experimental group treated with both CC and metformin [**11**]. The standardized difference was calculated by the following formula as being 0.96. This sample size was calculated to reject the null hypothesis that the failure rates for experimental and control subjects were equal with the probability (power) 90%. The Type I error probability (alpha) associated with this test of this null hypothesis is of 0.05. A line was drawn in the nomogram from 0.96 to the power axis at 0.9, and 42 subjects were found from the intersection with the central axis, at the 0.05 level of the study.

**Fig. 1:**
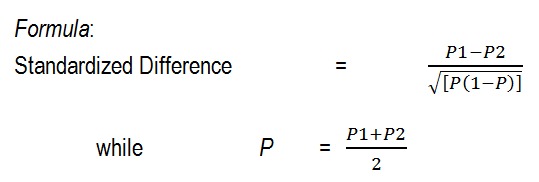
Formula

The first 42 patients diagnosed with PCOS were enrolled. The diagnosis was made based on the presence of two of the following three criteria:

 1. polycystic ovaries (either 12 or more peripheral follicles or increased ovarian volume (greater than 10 cm3)

 2. oligo- or anovulation (irregular cycles, amenorrhea)

 3. clinical and/or biochemical signs of hyperandrogenism (Acne, hirsutism, voice changes, clitoromegaly) 

 PCOS was only diagnosed after excluding other etiologies like thyroid dysfunction, congenital adrenal hyperplasia, hyperprolactinaemia, and androgen-secreting tumors. The baseline screening tests were done, e.g., thyroid function tests, a serum prolactin and free androgen index (total testosterone divided by sex hormone binding globulin (SHBG) × 100 to give a calculated free testosterone level). In cases of clinical evidence of hyperandrogenism and total testosterone > 5 nmol/l, 17-hydroxyprogesterone was done to exclude androgen-secreting tumors. Male factor was kept normal in both groups by making semen analysis to rule out male infertility. Moreover, females with recent history of pelvic inflammatory disease were not selected. Hysterosalpingogram was done to exclude tubal factor.

 The females were divided into group A and group B (21 subjects in each) by randomization for management with CC along with metformin and CC alone, respectively. The randomization was done by opening sequentially numbered opaque envelopes containing cards stating the management protocols.

 The group A received 500mg of metformin three times a day, continuously (the same dose), from the first cycle for six months, or until pregnancy was confirmed. In both groups, CC was started with a dose of 50 mg from the 2nd day of the menstrual cycle until day 6 for a total of 5 days. The dose of CC was increased to 100 mg during the second cycle and 150 mg in the third cycle, and the same dose of 150 mg was kept for the remaining three cycles. 

 With ovulation, the dose of clomiphene citrate was unaltered in both groups. Professionals and regulation of menstrual cycles followed all females for six cycles for evidence of ovulation as detected by follicle tracking (ovarian volume, size in mm and number of follicles) on ultrasonography. Dominant follicles confirmed ovulation on the 12th day with absent follicles on the 16th day. Confirmation of Conception was done by positive urine pregnancy test in those women who did not menstruate. In females who became pregnant, metformin was continued for up to 08 weeks of pregnancy. Clinical pregnancy was confirmed by a gestational sac detection on ultrasonography. 

 Data were analyzed by using SPSS version 16 (SPSS Inc., Chicago, IL, USA) and subjected to descriptive analysis, e.g., number, percentage. Chi-square test (2×2 contingency table) was applied to categorical variables.

### Ethical Issues

 The Institutional review board of the Hospital granted us permission to con¬duct this study and we declared that we have no financial or personal relationship(s), which may have inappropriately influenced us in writing this paper.

 A written formal informed consent from all the study participants was taken after they have been made aware of the study’s procedure. 

## Results

The subjects’ mean ±SD age (years) from group A was insignificantly higher than of group B, i.e., 32±3.5 vs. 31.3±2.9; p>0.05. Amenorrhea was common in group A 6(28.6) while the females in group B suffered more frequently from oligomenorrhea, e.g., 17(88.6). More than 50% females in both groups had a body mass index of >25.

Group A achieved a higher rate of regular cycles than group B (71.4% vs. 38.1%; p=0.03) while the ovulatory response was also found higher in group A than in group B (76.2% vs. 38.1%; p=0.021). Similarly, more females were found to have conceived by a confirmation through the urine pregnancy test in group A than in group B (66.6% vs. 28.6%, p=0.01), as well as group A had a higher conception rate confirmed on the presence of the gestational sac by ultrasound (61.9% vs. 28.6%; p=0.03). 

 Group A 60% females had complained of loss of appetite and 18% had nausea and vomiting, but none of them discontinued therapy. There were no cases of hyperstimulation either in the group A or B. No teratogenic effects were observed in patients who conceived after the treatment with metformin for ovulation induction. No twin pregnancy was found in neither of the two groups.

**Table 1:** Characteristics of Subjects

Variables	Group A	Group B	Difference (p-value)
Age (Mean ±SD)	32±3.5	31.3±2.9	NS
Oligomenorrhea	15(71.4)	17(88.6)	NS
Amenorrhea	6(28.6)	4(11.4)	NS
Hirsutism	18(85.7)	16(76.1)	NS
Acne	11(52.4)	8(38.1)	NS
BMI >25	14(56.7)	15(71.4)	NS
SD, Standard deviation; BMI, Body mass index; NS, Non-significant Data has been expressed in n(%)

**Table 2:** Subjects’ Outcome

Variables		Group A (n=21)	Group B (n=21)	p-value
Menstrual Cycle				
Menstrual Cycle regularity	Regular	15(71.4)	8(38.1)	0.03
	Irregular and/ Amenorrhea	6(28.6)	13(61.9)	
Ovulatory Responses				
Ovulation	Yes	16(76.2)	8(38)	0.012
	No	5(23.8)	13(61.9)	
Conception Confirmed on Urine Pregnancy Test				
Conception	Yes	14(66.6)	6(28.6)	0.013
	No	7(33.3)	15(71.4)	
Ultrasound Demonstration of Gestation Sac				
Conception	Yes	13(61.9)	6(28.6)	0.03

## Discussion

Polycystic ovarian syndrome was commonly treated by CC and gonadotropins, but this mechanism has given a new direction towards its management with insulin sensitizing drugs that give promising results and higher success rates of ovulation as well as pregnancy, as compared with CC [**12**]. 

 A major development in the clinical endocrinology happened with the discovery that metformin is effective in ovulation induction in women with PCOS [**11**]. Placebo-controlled studies, as mentioned by Cochrane review, obviously indicated that metformin is superior to placebo for inducing ovulation in infertile women with PCOS, with an odds ratio of 3.9 (confidence interval 2.3– 6.7) [**13**]. 

 Our study can be compared to a number of studies done at an international level. A study was carried out at Jordan showing the effect of metformin along with CC, as compared with placebo plus CC, on the ovulation and pregnancy rates in clomiphene citrate-resistant women with PCOS. Increased rates of ovulation (68.6% versus 25%, p<0.05) and pregnancy (56.3% versus 16.6%, p<0.05) were found in the metformin-clomiphene citrate group vs. placebo-clomiphene citrate controls. However, ovarian hyperstimulation in placebo-clomiphene citrate group was observed [**14**].

 A controlled trial was conducted in India to find out the occurrence of ovulation and pregnancy in infertile PCOS subjects who receive CC alone and a combination, e.g., metformin + CC. The metformin + CC resulted in a higher rate of ovulation than CC alone group (P = 0.0016). The pregnancy rate was of 8% with CC and of 24% with metformin + CC group. Loss of appetite was found in 80% of females of the study group, while 24% had nausea and vomiting. Moreover, no case of hyperstimulation was seen in either group. Finally, it was concluded that this combination should be initiated early in the course of treatment [**15**].

 Vandermolen et al., tried to determine whether metformin treatment increased the ovulation and pregnancy rates in response to CC in women with PCOS who were resistant to CC alone through a randomized, double-blind, placebo-controlled trial. Higher rates of ovulation as well as conception in metformin + CC vs. placebo + CC group were found, e.g., (75% vs. 27%) and (55% vs. 7%), respectively [**11**]. 

 Study of Zain et al., compared metformin alone, CC alone, and metformin + CC in three groups of subjects suffering from PCOS and found the highest success rate of ovulation of 68.7% and conception (21.1%) in metformin + CC group. However, CC should be used as the first line therapy in these females as they concluded [**16**].

 Contrary to the study of Zain et al., Neveu et al., found nearly similar ovulation rates among PCOS females treated with metformin alone and metformin + CC group. Furthermore, conception rates were also not different in the three groups, e.g., metformin alone, CC alone, and combination group. It was suggested that metformin could be used first for ovulation induction in females with PCOS despite their weight and insulin levels because of its efficacy and known safety profile [**17**]. 

 Decision-analytic model by Jungheim & Odibo, comparing the same three treatment strategies using probability estimates, derived after reviewing literature and sensitivity analyses performed on the baseline assumptions. Contrary to the studies of Neveu et al. & Zain et al., it was finalized that the combination therapy should be the first line therapy [**18**].

## Conclusion

A combination of metformin and clomiphene citrate significantly regulated the menstrual cycle and increased the ovulation and conception rates in study patients without complications, so, we prefer this combination therapy as first line therapy. 
